# Case report: a rare concurrence of dense deposit disease in an adolescent patient with IgA nephropathy

**DOI:** 10.1186/s12887-025-05415-z

**Published:** 2025-01-20

**Authors:** Jian-Hui Zhang, Hong-Ping Yu, Ying Chen, Qian Chen, Xiao-Ling Zheng, Jie-Wei Luo, Li Zhang

**Affiliations:** 1 Department of Traditional Chinese Medicine, Shengli Clinical Medical College of Fujian Medical University, Fujian Provincial Hospital, Fuzhou, China; 2 Department of digestive endoscopy, Fuzhou University Affiliated Provincial Hospital, Fuzhou, China; 3 Department of nephrology, Fuzhou University Affiliated Provincial Hospital, Fuzhou, China; 4 Shengli Clinical Medical College of Fujian Medical University, Fujian Provincial Hospital, no.134 East Street, Fuzhou, 350001 China

**Keywords:** Dense deposit disease, IgA nephropathy, Pathological complications, Case report

## Abstract

**Background:**

Dense deposit disease (DDD) is a rare renal disorder major affecting adolescents and children, characterized by an absence of distinctive clinical symptoms. Its coexistence with other renal conditions complicates both diagnosis and treatment in clinical practice.

**Case presentation:**

We described a 15-year-old male adolescent presenting with nephrotic syndrome as the initial manifestation, with urinalysis indicating significantly elevated protein and erythrocytes. Unexpectedly, the renal pathological biopsy of the patient exhibited strong positivity for both IgA and C3, characterized by petaloid deposition of C3 along glomerular capillary loops and the glomerular mesangial region, as well as linear deposition in Bowman’s capsule and portions of the renal tubular basement membrane. Consequently, the patient was diagnosed with both DDD and IgA nephropathy. The presence of both in a single patient may result in more intricate pathogenic pathways.

**Conclusions:**

This specific case elucidates the pathological characteristics of both diseases while investigating the intricate connections and lesion correlations that may occur between them, offering novel insights into their pathogenesis.

## Introduction

Dense deposit disease (DDD) is an uncommon renal disorder linked to anomalies in the complement bypass pathway, exhibiting an annual incidence of 1–2 cases per million individuals. It predominantly affects children and adolescents and has a dismal prognosis, with approximately 50% of patients advancing to end-stage renal disease (ESRD) within a decade [1]. Clinical manifestations encompass different degrees of hematuria, proteinuria, hypertension, and renal failure. Laboratory tests indicate persistent or recurrent low-complement C3 in the blood [2], with immunofluorescence revealing C3 deposition in the glomerular basement membrane and mesangial zone, accompanied by minimal or absent immunoglobulin deposition [3]. IgA nephropathy is a glomerulonephritis distinguished by the deposition of IgA immunoglobulins in the glomerular mesangial region. This illness may also present with C3 deposition in the same area and exhibit clinical manifestations akin to DDD, including hematuria, hypertension, or renal failure [4]. The pathological complexity and the challenges in diagnosis and treatment are markedly heightened when both diseases coexist in an individual. We present a rare renal pathological comorbidity: DDD in conjunction with IgA nephropathy, a case that illustrates the characteristic pathological symptoms of both conditions while also elucidating the intricate pathological link that may exist between them.

## Case presentation

The patient was a 15-year-old male adolescent who exhibited symmetrical swelling of the bilateral eyelids and lower extremities for a duration of three weeks, accompanied by significant frothy urine; however, urine volume remained normal, there was no visible hematuria, and the patient did not present with rash, fever, or musculoskeletal pain. The physical examination indicated elevated blood pressure of 160/112 mmHg. Routine urinalysis demonstrated a significant increase in protein and microscopic hematuria (Table 1). Blood biochemistry tests revealed a reduction in albumin and a substantial increase in plasma lipids, while the glomerular filtration rate remained normal. The patient had reduced levels of complement C3 and IgG; nevertheless, no clinically relevant alterations were observed in blood counts, coagulation, serum antibodies, or thyroid function (Table 1). The ultrasound of the urinary system revealed cortical echo enhancement in both kidneys.

**Table 1:** Clinical and biochemical characteristics in the patient

Test item		Normal value	Test item		Normal value
**Blood test**			**Coagulation function**		
WBC (×10^9^/L)	8.87	4.1 ~ 11.0	TT (sec)	17.5	14 ~ 21
RBC (×10^12^/L)	4.48	4.5 ~ 5.9	APTT (sec)	28.5	23.3 ~ 32.5
Hb (g/L)	131	129 ~ 172	PT (sec)	11.5	9.8 ~ 12.1
PLT (×10^9^/L)	298	150 ~ 407	FIB (g/L)	2.8	1.7 ~ 3.5
**Urine tests**			FDP (ug/mL)	4.25	0 ~ 5
Protein	+++	(-)	D-D(mg/L)	1.02	0 ~ 0.55
24 h-urinary proteins (g/24 h)	8.218	0.000 ~ 0.140	**Serum antibody**		
Glucose	(-)	(-)	PR3-ANCA	(-)	(-)
RBC (/u L)	1258	0 ~ 17	MPO-ANCA	(-)	(-)
WBC (/u L)	37	0 ~ 28	ANA	(-)	(-)
Urinary cylinder	(-)	(-)	Anti-dsDNA antibody	(-)	(-)
**Biochemical indexes**			HBsAg	(-)	(-)
TP (g/L)	44	65 ~ 85	Anti-PLA2R (RU/mL)	<5.00	<14.00
ALB (g/L)	20	40 ~ 55	**Thyroid function index**		
GLB (g/L)	24	20 ~ 40	TSH (mIU/L)	4.88	0.38 ~ 5.33
BUN (mmol/L)	3.8	1.8 ~ 6.4	FT3(pmol/L)	3.14	3.53 ~ 7.37
Scr(µmol/L)	66	21 ~ 77	FT4(pmol/L)	9.56	7.98 ~ 16.02
UA (umol/L)	288	208 ~ 428	**Humoral immune function test**		
LDH (U/L)	298	120 ~ 250	IgG (g/L)	2.55	7.00 ~ 16.00
TC (mmol/L)	10.03	<5.2	IgM (g/L)	1.600	0.400 ~ 2.300
TG (mmol/L)	2.94	<1.7	IgA (g/L)	2.340	0.700 ~ 4.000
HDL-C (mmol/L)	1.48	1.00 ~ 1.70	Complement C3 (g/L)	0.87	0.90 ~ 1.80
LDL-C (mmol/L)	7.30	1.30 ~ 3.60	Complement C4 (g/L)	0.30	0.10 ~ 0.40

*WBC* White blood cell count, *RBC* Red blood cell count, *Hb* Hemoglobin, *PLT* Platelet count, *TP* Total protein, *ALB* Albumin, *GLB* Globulin, *BUN* Serum urea nitrogen, *Scr* Serum creatinine, *UA* Uric acid, *LDH* Lactate dehydrogenase, *TC* Total cholesterol, *TG* Triglyceride, *HDL-C* High-density lipoprotein cholesterol, *LDL-C* Low-density lipoprotein cholesterol, *TT* Thrombin time, *APTT *Activated partial thromboplastin time, *PT* Prothrombin time, *FIB* Fibrinogen, *FDP* Fibrin degradation products, *D-D* D-dimer, *PR3* Proteinase 3, *MPO* Myeloperoxidase, *ANCA* Anti-neutrophil cytoplasmic antibodies, *ANA* Antinuclear antibody, *dsDNA* Double-stranded deoxyribonucleic acid, *HBsAg* Hepatitis b surface antigen, *PLA2R* Phospholipase A2 receptor antibody, *TSH* Thyroid Stimulating Hormone, *FT3* Free triiodothyronine, *FT4* Free thyroxine, *(-)* Negative

**Fig. 1:**
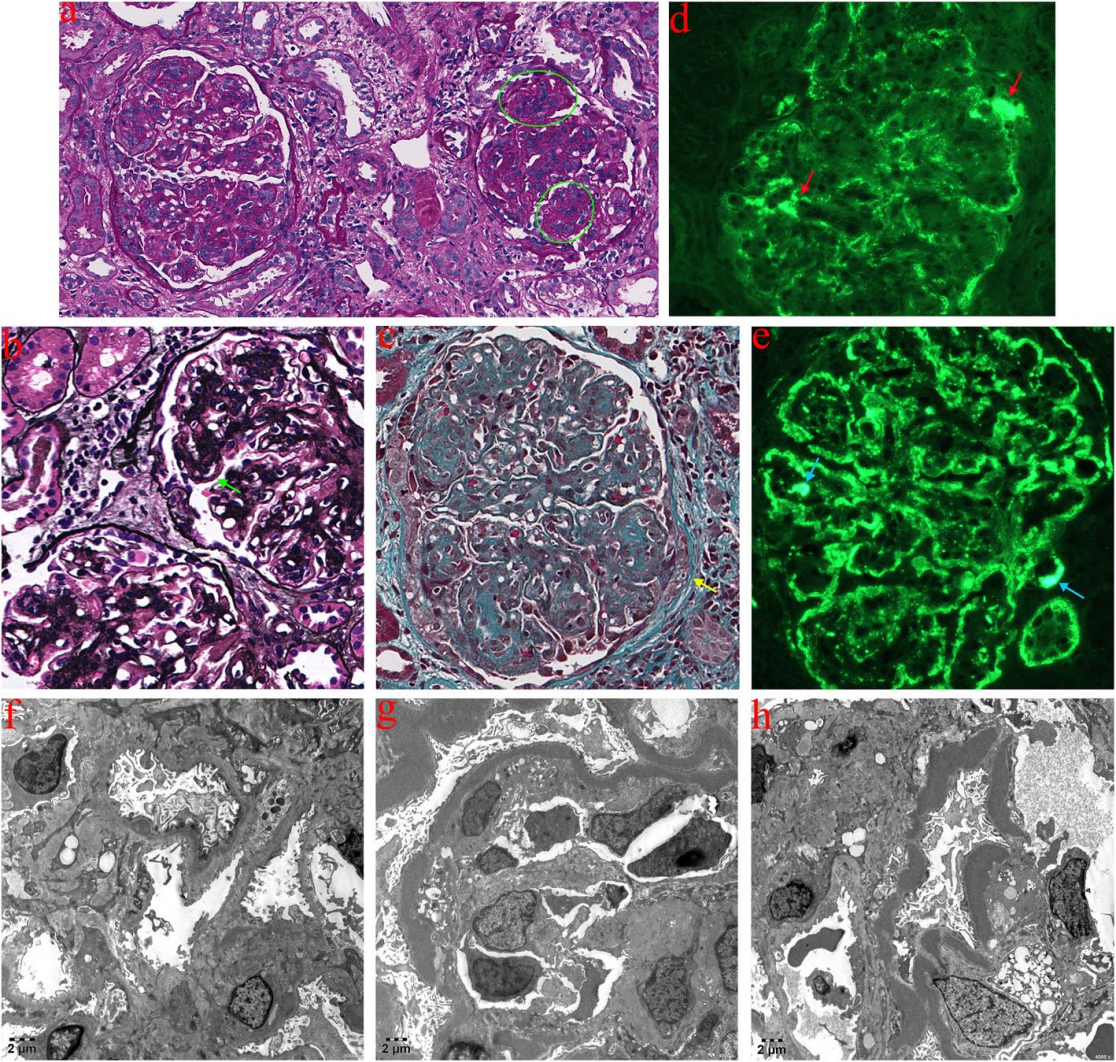
Renal pathological results for the patient. **a**-**c** Light microscopic observations reveal moderate to severe hyperplasia of glomerular mesangial cells (green circles) accompanied by nodular alterations, lobulated glomerular capillary loops, narrowing and occlusion of the capillary lumen, thickening of the basement membrane, segmental mesangial insertion, and double-track formation (indicated by the green arrows). Additionally, there is erythrophilic protein deposition in the mesangial region and subepithelial, along with the formation of small-cell fibrous crescentic bodies in certain areas (indicated by the yellow arrows), and mild fibrosis surrounding a few glomerular balloons (a, 150×; b, 300×; c, 300×). **d** Immunofluorescence of IgA, with red arrows indicating clumped deposition of IgA in the mesangial region and capillary loops. **e** Immunofluorescence of C3 reveals a blue arrow indicating C3 in the mesangial membrane region, alongside petal-like deposition of C3 along capillary loops and linear deposition in Bowman’s capsule and a portion of the renal tubule basement membrane. **f**-**h** Electron microscopy reveals segmental thickening of the glomerular basement membrane accompanied with diffuse fusion of podocyte foot processes, as well as high-density electron-dense deposits observed in the mesangial region, the dense layer of the basement membrane, and the wall of Bowman’s capsule

With informed agreement from the patient and his family, an ultrasound-guided renal biopsy was performed to determine the cause of nephrotic syndrome. Light microscopy of renal pathology demonstrates moderate to severe hyperplasia of glomerular mesangial cells, characterized by nodular alterations, lobulated glomerular capillary loops, and double-track formation. Additionally, there is deposition of erythrophilic protein in the glomerular mesangial region and subepithelial, along with the formation of partially visible small-cell fibrous crescentic bodies (Fig. 1a-c). Immunofluorescence exhibited strong positivity for both IgA and C3, characterized by petaloid deposition of C3 along glomerular capillary loops and the glomerular mesangial region, linear deposition in Bowman’s capsule and portions of renal tubular basement membranes (Fig. 1d), clumped deposition of IgA along the glomerular mesangial region and capillary loops (Fig. 1e), weak positivity for IgM, and negativity for IgG, C1q, Fib, ALB, IgG1, IgG2, IgG3, IgG4, PLA2R, and THSD7A. Ultrastructural analysis revealed segmental thickening of the glomerular basement membrane, extensive fusion of podocyte foot processes, and a high density of electron-dense deposits observed in the mesangial area, dense layer of the basement membrane, and the wall of Bowman’s capsule (Fig. 1f-h).

The patient was diagnosed with dense deposit disease and IgA nephropathy based on renal pathology findings. Initially, he had supportive therapy with valsartan and dapagliflozin to diminish proteinuria, alongside stringent blood pressure management utilizing diuretics and calcium channel blockers, as well as statins to ameliorate hyperlipidemia. Secondly, he underwent monthly impact therapy consisting of 800 mg cyclophosphamide in conjunction with 40 mg methylprednisolone daily, while also utilizing proton pump inhibitors and anti-osteoporosis medications to mitigate adverse effects. One week post-drug treatment, edema showed significant improvement. Three months later, urinary erythrocytes and protein levels were markedly reduced, the 24-hour urine protein measurement was 0.974 g, and the plasma albumin level was 41 g/L; blood pressure remained within normal limits, and the alteration of complement component C3 was minimal.

## Discussion

Dense deposit disease is typically diagnosed at an average patient age of 14 years [5]. The pathological characteristics of DDD consist of significant thickening of the glomerular basement membrane and the detection of high electron density deposits in the dense layer of the glomerular basement membrane observed under electron microscopy. Electron microscopy is an indispensable tool in diagnosing DDD, with distinctive alterations manifesting as ‘sausage-shaped’ or ‘Chinese calligraphy-like’ electron-dense deposits in the glomerular basement membrane and mesangial region [6]. In clinical presentation, DDD may show as acute nephritis, proteinuria, or nephrotic syndrome [7]. Light microscopy of the renal tissue in the majority of patients with DDD reveals characteristics of mesangial proliferative glomerulonephritis and crescentic glomerulonephritis [8]. The clinical presentation and pathological characteristics of the patient in our study were highly consistent with DDD, offering significant evidence for further investigation of pathological complications.

DDD has been linked to type II membranoproliferative glomerulonephritis, C3 convertase in membranoproliferative glomerulonephritis, and IgA in IgA nephritis, all of which are significant elements of the immune system that may result in autoimmune nephropathy [9]. The aberrant activation of the complement paracrine pathway in DDD leads to the persistent degradation of C3 into C3a and C3b, a reduction in serum C3 levels, and the formation of membrane attack complexes that accumulate in the glomerular basement membrane, resulting in renal damage; moreover, the deposition of IgA immune complexes not only initiates the complement paracrine pathway but also activates the lectin pathway [10]. It is worth noting whether the concurrent presence of this kidney pathological comorbidity is merely coincidental or indicative of a link between the two. Complement activation and immune complex deposition in DDD may intensify the inflammatory response and glomerular injury in IgA nephropathy. Abnormal complement system activation can directly harm glomerular cells and enhance the inflammatory response by recruiting inflammatory cells and releasing inflammatory mediators [11]. Conversely, IgA deposition of IgA nephropathy may compromise the integrity of the glomerular basement membrane and subsequently enhance the accumulation of electron-dense material in DDD. Furthermore, the concurrent presence of both may also involve more extensive immunomodulatory irregularities. For example, certain genetic or environmental variables may influence both the glycosylation alterations of IgA1 and the proper functioning of the complement system [12], hence elevating the risk of concurrent DDD and IgA nephropathy.

The primary objective of treatment for both DDD and IgA nephropathy is to regulate blood pressure and diminish proteinuria. Inhibitors of the renin-angiotensin-aldosterone system and sodium-glucose cotransporter protein 2 may serve as fundamental treatments for both IgA nephropathy and DDD; however, according to KDIGO guidelines, immunosuppressive therapies are applicable for patients with moderate to severe disease (characterized by proteinuria exceeding 1 g/day, hematuria, or diminished renal function persisting for at least 6 months) [13]. The patient exhibits clinical manifestations of nephrotic syndrome, characterized by small cell fibrous crescents. The treatment regimen included cyclophosphamide combined with glucocorticoids, to which the patient responded favorably, evidenced by a significant reduction in urinary red blood cells and proteins during follow-up. However, to ascertain the potential for long-term remission or recurrence, it is imperative to continuously monitor fluctuations in antibody levels and extend the duration of follow-up.

## Data Availability

No datasets were generated or analysed during the current study.
